# Bayesian Estimation of the True Bovine Brucellosis Prevalence in Vaccinated and Non-Vaccinated Ecuadorian Cattle Populations, and the Sensitivity and Specificity of a Competitive and Indirect ELISA Using a New Synthetic Antigen

**DOI:** 10.3390/microorganisms13010069

**Published:** 2025-01-02

**Authors:** Ana Dolores Garrido Haro, Margoth Yolanda Barrionuevo Samaniego, Paola Moreno-Caballeros, Alexandra Burbano-Enríquez, Verónica Alexandra Salas Torres, María Cristina Galante Mulki, Constance Wielick, Jorge Ron-Román, Claude Saegerman

**Affiliations:** 1 Agrocalidad Tumbaco, Quito 170903, Ecuador; ana.garrido@agrocalidad.gob.ec (A.D.G.H.); margoth.barrionuevo@agrocalidad.gob.ec (M.Y.B.S.); paola.moreno@agrocalidad.gob.ec (P.M.-C.); lidia.burbano@agrocalidad.gob.ec (A.B.-E.); veronica.salas@agrocalidad.gob.ec (V.A.S.T.); maria.galante@agrocalidad.gob.ec (M.C.G.M.); 2 Research Unit of Epidemiology and Risk Analysis Applied to Veterinary Science (UREAR-ULg), Fundamental and Applied Research for Animals & Health (FARAH) Center, Faculty of Veterinary Medicine, University of Liege, 4000 Liège, Belgium; cwielick@uliege.be; 3 Grupo de Investigación en Sanidad Animal y Humana (GISAH), Carrera Ingeniería Agropecuaria, Departamento de Ciencias de la Vida y la Agricultura, Universidad de las Fuerzas Armadas ESPE, Sangolquí 171103, Ecuador; jwron@espe.edu.ec

**Keywords:** bovine brucellosis, Ecuador, Bayesian approach, ELISA, test performance, synthetic oligosaccharides, vaccine

## Abstract

Bovine brucellosis (bB) is a zoonosis mainly caused by the *Brucella abortus* species in cattle. Bovine brucellosis can present with either a range of clinical symptoms, including spontaneous abortions in the last trimester of pregnancy, retained fetal membranes, and decreased milk production, or it can be asymptomatic. In Ecuador, vaccination against bB with S19 and/or RB51 is not mandatory and is the responsibility of the farmer. As serology is a convenient method for detecting antibodies against *Brucella*, evaluating the diagnostic performance and discriminative ability of such tests in various epidemiological settings is required. To estimate and compare the diagnostic sensitivity (Se) and specificity (Sp) of two screening tests, a new competitive (cELISA) and an indirect ELISA based on a new synthetic antigen (iELISA), a randomized, stratified, cross-sectional, serological survey was performed on the cattle population (3299 bovine sera from 223 farms) in continental Ecuador. A Bayesian approach was used to evaluate the two tests by estimating their respective diagnostic Se and Sp, as well as the true prevalence of bB in different sub-populations (non-vaccinated, vaccinated with S19 or RB51). The Se of both tests was similar across Bayesian models, with values around 94%. In contrast, the Sp of the iELISA, ranging between 97 and 98%, was significantly higher than that of the cELISA, which was approximately 94–95%. The true prevalence of bB was 1.63% (95% CrI: 0.56–2.54) in non-vaccinated cattle, decreased to 0.97% (95% CrI: 0.005–2.54) in S19-vaccinated cattle and was 2.75% (95% CrI: 0.50–5.32) in RB51-vaccinated cattle. The results of this study suggest that, with similar Se and higher Sp, the iELISA based on an innovative synthetic antigen (which is more standardizable) should be recommended as a possible screening test for bB in Ecuador. Also, the proposed approach suggests insights into the quality of the vaccination campaign and highlights the need for refining the Ecuadorian national brucellosis control program.

## 1. Introduction

Bovine brucellosis (bB) is a worldwide zoonotic disease, mainly caused by *Brucella abortus*, a facultative intracellular pathogen mostly associated with cattle, its natural or primary host [1,2]. Cattle become infected through close contact with infected animals, uterine secretions or aborted fetuses, vertical and sexual transmission, and by ingesting contaminated food, milk/colostrum, forage and water [2,3]. The disease causes substantial economic losses due to abortion, mostly in the last trimester of pregnancy, mastitis and reduced milk production in female animals, and orchitis and epididymitis in male animals. Infertility can occur in both male and female animals [4]. The annual loss due to bB has been estimated at USD 600 million in Latin America [5]. In addition, a 20–30% decrease in milk production has been estimated in bB-affected herds [6,7]. Economic losses in the livestock herds of San Pedro de Suma, located in the province of Manabí in the Coastal region, were estimated to range between USD 1922 and 3843 at the herd level [8].

In humans, bB is an occupational disease mainly transmitted through close contact with contaminated placenta, urine, excrement, blood and aborted fetuses. It predominantly affects workers who handle domestic ruminants, such as veterinarians, veterinary assistants, farmers, slaughterhouse workers, butchers, and laboratory workers [9,10,11].

Bovine brucellosis has been reported in Latin America since the first decade of the 20th century and remains a major zoonosis despite ongoing campaigns for its control. Ecuador has four natural regions: the Coast, the Highlands, the Amazon and the Galapagos. The Galapagos Islands, which have only a small cattle population, have been recognized as free of brucellosis without vaccination [12]. However, bB remains endemic in continental Ecuador [13].

In Ecuador, the Phytosanitary and Zoosanitary Regulation and Control Agency (Agencia de Regulación y Control Fito y Zoosanitario—Agrocalidad) is responsible for the national bB control program, which began in 2008. The program is based on the voluntary vaccination of females with S19 and/or RB51 strains, serological diagnosis (using the Rose Bengal, iELISA, and cELISA), the culling of positive animals and the certification of herds as free of bB [14]. Herds with brucellosis-free certification receive a bonus of USD 0.01 per liter of milk from pasteurizers [15].

In cattle, the clinical presentation of bB is diverse and not pathognomonic. The isolation and identification of *Brucella* spp. is considered as the reference standard diagnostic method; a positive test result provides an unequivocal diagnosis of brucellosis [16,17]. However, these methods are not always feasible in developing countries for brucellosis diagnostic investigations (necessitating a BSL3 laboratory and well-trained personnel). Therefore, diagnosis is frequently based on serological method(s) used alone or in combination, in serial or in parallel, which, while not perfect, are recommended by the WOAH for trade purposes [9,17,18]. Since serology is the most convenient method for detecting antibodies against *Brucella*, evaluating the diagnostic performance and discriminative ability of these tests in various epidemiological settings is crucial for effective bB control. To achieve this, WOAH accepts the Bayesian approach for estimating the diagnostic sensitivity (Se) and specificity (Sp) of these tests [16].

Based on traditional diagnostic tests (Rose Bengal and SAT-EDTA tests), the prevalence of bovine brucellosis in Ecuador was previously estimated at 1.6% in small and medium-scale cattle herds [13]. Currently, the diagnostic tests routinely used in Ecuador include the Rose Bengal test and other commercially available iELISA and/or cELISA. Competitive ELISA (cELISA) has been recognized for testing bB in different animal and human species and is generally considered as highly specific, while indirect ELISA (iELISA) is considered highly sensitive [2,9]. For both ELISA tests, a critical point is the difficulty of maintaining a consistent quality of the *Brucella* lipopolysaccharide coated on the assay plate. The recent discovery of synthetic oligosaccharide antigens, which represent the capping M epitope elements of *Brucella* O-polysaccharides, offers an opportunity to more easily standardize the quality of the antigen used and to increase the specificity of the brucellosis serodiagnosis [19,20].

The aims of this study were to estimate the characteristics (Se and Sp) of a cELISA and an iELISA based on a new synthetic antigen, using a Bayesian approach, and to determine the true prevalence of bB in continental Ecuador in different epidemiological settings (vaccinated and non-vaccinated cattle).

## 2. Materials and Methods

### 2.1. Study Area

**Figure 1:**
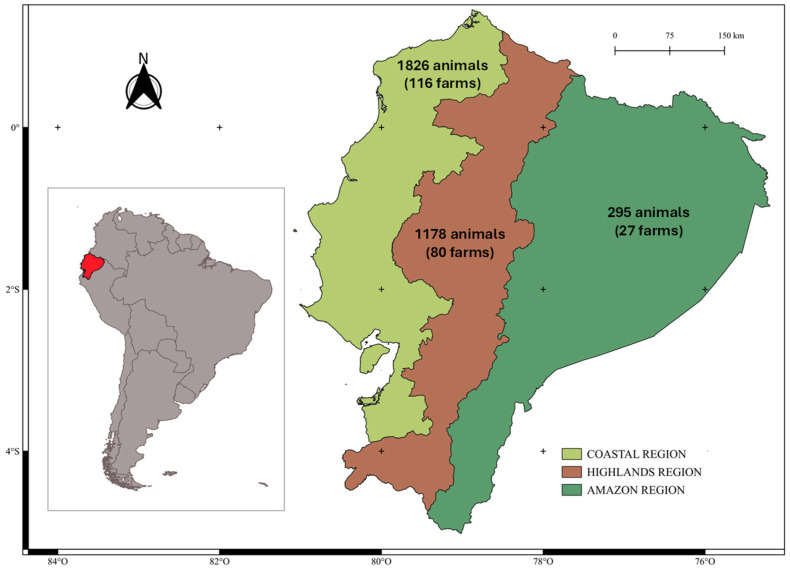
Geographic regions of continental Ecuador and sampling.

Ecuador covers 281,341 km^2^ and is divided into four regions, which include 24 provinces [21]. The three continental regions are the Coastal region in the western part, the Highlands in the central part, and the Eastern region (i.e. Amazonia). The fourth region constitutes the Galapagos Islands (Figure 1).

In Ecuador, the agricultural sector contributes to the gross national product by 8% [22], and 5.7 million liters of milk are produced daily at the national level, providing employment for 1,140,000 Ecuadorians [23]. According to the national cadaster, Ecuador has 4,525,183 cattle originating from 285,579 farms, with around 41% of the animals in the Coastal region, 49% in the Highlands, and the remainder in Eastern region [24].

The Ecuadorian climate varies by region due to differences in altitude, proximity to the equator, and proximity to the Pacific Ocean [25]. The Coastal region has a wet season from December to May and a dry season from June to November, with temperatures ranging from 23 °C to 26 °C. The climate in the Highlands (Andes Mountains) is cold and rainy from November to April and dry from May to October, with temperatures ranging from 13 °C to 18 °C. In the eastern Amazon region, the climate is rainy and humid between January and September and dry between October and December, with temperatures ranging between 23 °C and 26 °C. The Galapagos Islands have a temperate climate with temperatures ranging from 22 °C to 32 °C.

### 2.2. Sampling Protocol and Study Design

To determine an adequate sample size for the assessment of the bB serological status in Ecuadorian cattle, a two-stage sampling design was used. In the first stage, at the herd level, the parameters included a confidence level of 95%, a test Se and Sp of 95%, and an estimated disease prevalence of 15% with 5% precision. In the second stage, at the animal level, the parameters were a confidence level of 95%, a test Se and Sp of 95%, and estimated disease prevalence of 10% with 5% of precision. To minimize the occurrence of false positive serological reactions (FPSR) due to brucellosis vaccination in Ecuador, 24-month-old female bovines were sampled (N = 3299 bovines from 223 herds). Studies have shown that antibodies detected by traditional serological tests following vaccination with the S19 vaccine in young animals decrease over time, and becoming negligible after 24 months [9,26]. For the RB51 vaccine, due to its minimal production of O-chain, it usually does not induce antibodies against the O-chain in quantities measurable by traditional serological tests [27]. However, for both vaccines, detectable antibodies can be measured using more sensitive tests such as ELISA [28,29].

The 23 provinces of mainland Ecuador were surveyed between May and June 2018. Blood samples were collected in tubes without anticoagulant by puncturing the coccygeal vein of each animal. The samples were transferred to Agrocalidad laboratories, maintaining the cold chain at 4 °C to 8 °C. To separate the blood serum, the samples were centrifuged for 5 min at 5000 rpm. The serum samples were stored at 4 °C to 8 °C until analysis in Agrocalidad‘s serology laboratory.

This survey was designed and performed by the Agencia de Regulación y Control Fito y Zoosanitaria (Agrocalidad), a public agency under the Ministry of Agriculture and Livestock of Ecuador. The blood samples were collected with the prior consent of the herd owners and did not involve any cost to them.

### 2.3. Diagnostic Tests

To reveal the presence of anti-bB antibodies among the sampled cattle, two new diagnostic tests were used in parallel: the first was a competitive ELISA (cELISA), and the second was an indirect ELISA (iELISA) using a synthetic antigen.

#### 2.3.1. Competitive ELISA (cELISA)

Greiner 762021 F8 microplates (Greiner, Kremsmünster, Austria) were coated overnight at 4 °C with a *Brucella abortus* lipopolysaccharide (LPS) preparation provided by the Belgian Sciensano Institute. The optimal coating dilution of the LPS solution was determined using checkerboard titration by varying both the dilution of the LPS and the conjugate. Each serum sample was tested in duplicate. Briefly, the serum samples were diluted at 1:2 for testing. For each assay, negative and positive controls were included. Fifty µL of diluted sample were used. After a 120 min incubation period at 37 °C ± 3 °C, the plates were washed with a washing solution before a conjugate (Monoclonal A76-12B12 coupled to the horseradish peroxidase) was added. Following a second incubation period of 30 min at 37 °C ± 3 °C, the plates were revealed by adding 3,3′,5,5′-tetramethylbenzidine (TMB) and incubating for 10 min at room temperature (21 °C ± 3 °C). Finally, the reaction was stopped by adding a blocking solution. The optical densities (OD) in the microwells were read using a Thermo Scientific™ Multiskan FC, Version ESW 1.01.16, Serial No. 357-704865 plate reader. Software version: SkanIt Software for Microplate Readers RE, ver. 6.0.2.3, with a 450 nm absorbance filter. Results must be read fairly soon after the stopping solution has been added (10 min), since the chromogen may crystallize in wells with strong signals and distort the results accordingly. The results were expressed as % inhibition using the following equation:

(1) $$\frac{OD\;neg-OD\;sample}{OD\;neg}\times 100$$

where OD is the optical density, and neg is the negative control sample.

**Figure A1:**
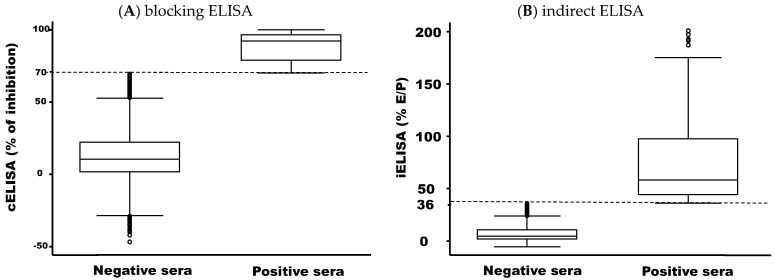
The cut-off values of the competitive ELISA (**A**) and the indirect ELISA with the synthetic antigen (**B**) were calculated based on the official reference sera provided by the Belgian federal reference laboratory Sciensano. The cut-off values were determined as 70% inhibition and 39% sample/positive (S/P) control ratio for the cELISA and the iELISA, respectively (discontinued horizontal line).

The cut-off value of the cELISA kit was calculated to be in line with the official reference serum provided by the Belgian federal reference laboratory Sciensano and was determined as 70% inhibition (see also Figure A1).

#### 2.3.2. Indirect ELISA Using a Synthetic Antigen (iELISA)

A synthetic antigen composed of M-tetrasaccharides coupled to bovine serum albumin (BSA) was supplied by the Animal and Plant Health Agency (APHA Scientific, Addlestone, UK) [19]. The M-epitope is an α1,3-linked D-Rha4NFo disaccharide. The tetrasaccharide consists of the disaccharide structure with an additional O-polysaccharide (OPS) sugar unit attached to each end. This maintains the unique *Brucella* OPS epitope, while incorporating side structures that increase the size of the antibody epitope. Multiple tetrasaccharides are conjugated to a protein carrier (BSA) to enable passive adsorption onto ELISA plates.

Greiner 762021 F8 microplates were coated overnight at 4 °C with 100 µL of the synthetic antigen at a concentration of 2.5 μgr/mL. The plates were then saturated with a casein hydrolysate solution, dried, and stored in aluminum bags with desiccant. Each serum sample was tested in duplicate. Briefly, the serum samples were diluted 1:100 for testing. For each assay, negative and positive controls were included. Twenty microliters of sample were used. After a 60 min incubation period at room temperature (21 °C ± 3 °C), the plates were washed with a washing solution, and a protein G-conjugated antibody coupled to the horseradish peroxidase was added. Following a second 60 min incubation at room temperature (21 °C ± 3 °C), the plate was revealed with 3,3′,5,5′-tetramethylbenzidine (TMB) for 10 min at room temperature and the reaction was stopped by adding a blocking solution. The optical densities (OD) in the microwells were read using a Thermo Scientific™ Multiskan FC, Version ESW 1.01.16, Serial No. 357-704865 plate reader. Software version: SkanIt Software for Microplate Readers RE, ver. 6.0.2.3, with an absorbance 450 nm filter. Results must be read soon after the stopping solution has been added (10 min) since the chromogen may crystallize in wells with strong signals and distort the results accordingly. Results were calculated as the ratio of the optical density (OD) of the serum sample (S) to the OD of the positive control (P) and expressed as a percentage (% S/P):

(2) $$\frac{OD\;sample}{OD\;pos}\times 100$$

where OD is the optical density, and pos is the positive control sample.

The cut-off value of the iELISA kit, based on the use of the synthetic antigen, was calculated to be in line with the official reference provided by the Belgian federal reference laboratory Sciensano and was determined as 36% S/P (see Figure A1).

#### 2.3.3. Repeatability and Reproducibility of the cELISA and iELISA Used

The intra-assay repeatability was estimated using a weak sample (internal reference serum from Sciensano, diluted 1:4 for cELISA and 1:400 for iELISA), which was tested 288 times across three different plates. The intra-laboratory reproducibility was evaluated using four dilutions (1/4; 1/8; and 1/16 for cELISA; and 1/50; 1/100; 1/200; and 1/400 for iELISA) of the same sample. These dilutions were tested in triplicate on six separate occasions, across different days, and by two operators. Acceptable coefficients of variation for the repeatability and for the reproducibility were fixed as <10% and <15%, respectively.

#### 2.3.4. Assessment of Agreement Between the Tests

The two tests were compared using concordance analysis to assess their agreement. First, the overall level of agreement was measured using the Kappa coefficient [30]. Next, the agreement was evaluated in terms of indices of positive and negative agreement [31], which represent the observed agreement proportions for positive and negative test results, respectively. Confidence intervals were calculated according to the method of Graham and Bull [32]. Using a ‘two-by-two’ contingency table (Table 1), the indices of positive agreement (p_pos_) and negative agreement (p_neg_) were calculated as follows:

(3) $$p_{pos}=\frac{2a}{2a+b+c}$$

(4) $$p_{neg}=\frac{2d}{2d+b+c}$$

where p_pos_ and p_neg_ were the indices of positive and negative agreement, respectively (parameters a, b, c and d are detailed in Table 1).

### 2.4. Bayesian Analysis

The methodology developed by Sanogo et al. [17,33] was applied to estimate the sensitivity and specificity of the ELISA assays, as well as the true prevalences. The imperfect Se and Sp of diagnostic tests can lead to misclassification of tested animals [17]. Therefore, to estimate test characteristics under field conditions, Se and Sp values should be derived from prior data or, in the case of absence, from expert opinion (prior distribution) [33]. Bayesian statistics allow the determination of the likelihood function by incorporating prior information about the parameters into the analysis alongside the observed data. By using Bayes’ theorem, both the prior distribution and the likelihood function yield a posterior distribution, representing updated knowledge [33,34]. In the context of epidemiology and diagnostic testing, Berkvens et al. (2006) [35] described a Bayesian approach using probabilistic constraints to estimate true disease prevalence and diagnostic test characteristics when multiple diagnostic tests are applied to a set of individuals in a population.

#### 2.4.1. Estimation of Test Sensitivity and Specificity and True Prevalence

In the absence of a ‘gold standard’, a Bayesian approach was used to evaluate the performance of the cELISA and the iELISA assays by estimating their respective Se and Sp [34,35] (Table A1 and Table A2). Given that both tests are based on antibody detection, they can be considered conditionally dependent, i.e. the results of the two tests for a given animal are correlated [17]. Consequently, a Bayesian model was developed considering the correlation between the tests in both infected and non-infected animals. This approach allowed for the inclusion of both field data and prior expert information into a single model to estimate the characteristics of the tests (Se and Sp) as well as the true prevalence and their respective 95% credibility intervals (CrI).

Based on the scientific literature, realistic prior information regarding prevalence was between 0 and 10%, translated as a uniform distribution between 0 and 0.1 [13,36,37]. For the estimation of Se and Sp, data were synthesized from a review paper and a book chapter. For cELISA and iELISA, the Se was between 0.95 and 1, translated as a beta distribution characterized by an alpha of 96 and a beta of 6. The Sp was represented as a uniform distribution comprised between 0.95 and 1) [2,9]. The model was established within WinBUGS using covariance [34,38] (Appendix D.1, Appendix D.2, Appendix D.3, Appendix D.4, Appendix D.5, Appendix D.6, Appendix D.7 and Appendix D.8 in Appendix D). Three parameters were monitored during the analysis: (i) the deviance information criterion (DIC), (ii) the effective number of estimated parameters (pD), and (iii) the Bayesian *p*-value [39]. In brief, the DIC and Bayesian *p*-value were used to check for conflicts between the prior information and the testing data results (i.e., based on the likelihood of observations). The DIC is a generalization of the Akaike Information Criterion (AIC) for a multinomial model [35] and its value should be positive and as low as possible. The pD of the model was used to assess the impact of the constraints. The optimal pD can be calculated using the following formula [35]:

(5) $$2^{n}-1$$

For the two tests, the optimal pD value is three.

The model used three chains, a ‘burn-in’ of 10,000 iterations, and an additional 10,000 iterations to obtain the posterior distributions. Trace plots were used in conjunction with autocorrelation plots to assess model convergence. If the trace plot indicated good mixing and the autocorrelation plot little or no correlation among samples, then convergence was considered achieved. If autocorrelations were still high after the first few lags, ‘thinning’ was applied, with the thinning coefficient determined by the number of lags at which autocorrelations significantly dropped to zero. A more formal test for convergence, the Brooks–Gelman–Rubin (BGR) statistic, was also used [40]. A model was considered of good quality (with appropriate priors) when the following criteria were present: good mixing of chains as indicated by the BGR statistic, a Bayes-*p* value close to 0.5, a pD value close to 3, and a posterior density distribution that was clearly identifiable (e.g., a distribution with a distinct shape).

#### 2.4.2. Priors and Sensitivity Analysis

The parameter estimates obtained using the Bayesian model with conditional dependence between tests can vary depending on the prior distributions used [34]. To assess the influence of the proposed prior distributions on the estimated parameters, a sensitivity analysis was performed using less informative priors [34,41]. For this analysis, a uniform distribution between 0 and 0.2 was used for prevalence, as bB prevalence varies considerably across Latin America with rates ranging from 0.5% to 10%. The Se was set at 50%, represented by a beta distribution with an alpha of 51 and a beta of 51, and the Sp was represented by a uniform distribution ranging between 0.5 and 1 (since tests with characteristics below 0.5 are generally not commercialized). For each set of alternative prior distributions considered for the parameters, the model was run with the same number of chains, and similar quality criteria were checked.

## 3. Results

### 3.1. Repeatability and Reproducibility of Both cELISA and iELISA Assays

The intra-assay repeatability (coefficient of variation, CV) was 6.03% and 7.02% for cELISA and iELISA, respectively, both of which meet the required criterion (CV < 10%). The intra-laboratory reproducibility ranged from 4.44 and 11.02 for cELISA and from 2.02% to 9.27% for iELISA, respectively, also meeting the required criterion (CV < 15%).

### 3.2. Serology

Considering the two-by-two contingency table (Table 1), cross-classified test results of 3299 random samples resulted in: a = 64, b = 117, c = 43, and d = 3075. A total of 107 (3.2%, a + c) serum samples tested positive with the cELISA assay, whereas 181 (5.5%, a + b) serum samples were positive using the iELISA assay. Both tests exhibited the same results for 3139 serum samples (95.15%, a + d).

### 3.3. Indices of Agreement Between Both cELISA and iELISA Assays

The cross-classified test results were used to calculate the indices of agreement between the two assays. Both assays showed the same result for 95.15% of the serum samples (95% CI: 94.36–95.86). The concordance between the two tests was estimated as moderate, with a Kappa coefficient of 0.42 (95% CI: 0.39–0.45). The agreement on positive results for both tests (*p_pos_*) was estimated at 44.44% (95% CI: 38.62–50.39), whereas the agreement on negative test results (*p_neg_*) was estimated at 97.46% (95% CI: 97.05–97.84). These indices suggest that the test outcomes for the animals are correlated.

### 3.4. Estimated True Prevalence, Test Sensitivity and Specificity in Different Settings

The estimation of true prevalence, as well as test Se and Sp, was performed using all animals (n = 3299; model 1) and in three different settings: non-vaccinated animals (n = 2506; model 2), vaccinated animals with S19 (n = 383; model 3) and vaccinated animals with RB51 (n = 392; model 4) (Table 2).

After some pre-testing and the application of a thinning coefficient of 100, all models used in this study appeared to converge, as indicated by properly mixed chains and autocorrelations equal to zero. The BGR plots also supported these findings. The estimated Bayesian *p*-values were 0.49, 0.49, 0.52 and 0.47 for models 1 to 4, respectively, indicating no particular issues with model fit (i.e., values close to 0.5). The pD values estimated from the multinomial probabilities were 2.88, 2.82, 2.58 and 2.62 for models 1 to 4, respectively, which were relatively close to the optimal value of 3. The DIC values were estimated at 23.92, 23.10, 16.11 and 17.68 for models 1 to 4, respectively, all of which were acceptable values (positive and as small as possible) (Table 3).

The estimated values of Se and Sp for both cELISA and iELISA are summarized in Table 2. The Se (around 94%) was similar across tests and models. In contrast, the Sp of the iELISA (approximately 97–98%) was significantly higher than that of the cELISA (around 94–95%), as indicated by distinct or minimally overlapping CrIs.

As an outcome of model 1, the overall true prevalence of brucellosis was estimated to be 1.50% (95% CrI: 0.36–2.47). Using models 2 to 4, the true prevalence was estimated in three different settings: 1.63% (95% CrI: 0.56–2.54) in non-vaccinated animals, 0.97 (95% CrI: 0.005–2.54) in S19-vaccinated animals, and 2.75 (95% CrI: 0.5–5.32) in RB51-vaccinated animals. These three true prevalences were significantly different (ordered regression analysis performed after exportation of the output data; *p*-value < 0.001).

### 3.5. Sensitivity Analysis

Using non-informative priors for prevalence, Se and Sp, the corresponding posterior estimates are summarized in Table 2. The validity criteria (i.e., BGR, Bayesian *p*-value (Bayse p), DIC, pD and density distribution) were met and are presented in Table 3. The results indicated that, using non-informative priors, (i) the estimated prevalence increased by approximately 1.5 to 2.5 times depending on the setting (all animals, non-vaccinated, S19 vaccinated, and RB51 vaccinated animals); (ii) the estimated Se decreased drastically, reaching around 50% for all animals (model M1-SA) and vaccinated groups (models M3-SA and M4-SA), and about 70% for non-vaccinated animals (model M2-SA); and (iii) the estimated Sp for models M1-SA to M4-SA was similar to that for models M1 to M4 with informative priors (Table 2).

The use of non-informative priors resulted in models with good mixing of the Markov chains (BGR), Bayesian *p*-values, and DIC values close to the optimal values and those obtained with models M1 to M4. However, for models M1-SA to M4-SA, the DIC was systematically higher compared to models M1 to M4. In addition, the density distributions of models M1-SA to M4-SA were less identifiable than the those of models M1 to M4 (Table A3). Consequently, models M2 to M4 were selected as the best models for estimation based on the different settings (non-vaccinated, S19 vaccinated, and RB51 vaccinated).

## 4. Discussion

The aims of this study were to estimate the true prevalence of bovine brucellosis (bB) in Ecuador and diagnostic characteristics of two ELISA tests, one competitive (cELISA) and one indirect with a new synthetic oligosaccharide antigen representing the capping M epitope elements of *Brucella* O-polysaccharides (iELISA) in non-vaccinated, S19 vaccinated and RB51 vaccinated settings [29]. This study, based on a large-scale cross-sectional serological survey, demonstrated that depending on the setting (model M1 to M4), different prevalences were observed. Both tests showed a similar Se (94%), but iELISA had a higher Sp compared to cELISA (approximately 2% higher). The overall agreement between cELISA and iELISA was moderate, with good agreement for negative results and moderate agreement for positive results.

In this study, 3299 bovines older than 24 months coming from 223 farms, originating from all 23 provinces of continental Ecuador, which is endemic for bB, were randomly sampled. The randomized large sampling induces representativeness and accuracy of the estimates. The estimation of the Se and Sp of a diagnostic test requires knowledge of the true disease status of the animals on which this assay is applied [33]. In absence of perfect tests (Se and Sp = 100%), a Bayesian approach is helpful to estimate the test Se, Sp and prevalence of bB (e.g., [42]). We used literature references for the estimation of the priors as true prevalence [36,37], Se and Sp of both cELISA and iELISA [2,9]. Using these priors (see Table 2) and using Bayesian modeling, we calculated posterior estimations of the true prevalence and the Se and Sp of the two tests in different settings. The difference in Sp between both ELISA assays explains the low positive agreement between those tests. In general, the Sp is better with a cELISA [2,9]; however, in this case, the iELISA exhibits a higher Sp, and this is attributed to the nature of the synthetic antigen used and constituted by capping M epitope elements of *Brucella* O-polysaccharides that are unique to *Brucella* [26]. The iELISA with the synthetic antigen gives two advantages: it is more easily standardizable and gives less false positive serological reactions due to its higher specificity [29]. Furthermore, the reduction in false positive results will lead to a decrease in the costs associated with unnecessary slaughter for Ecuadorian authorities. Given these test characteristics, the iELISA based on innovative synthetic antigens can be proposed as alternative screening test for the national bovine brucellosis control program in Ecuador in replacement to the current tests routinely used (Rose Bengal test, other commercialized iELISA and/or cELISA).

After the sensitivity analysis using non-informative priors, M2 to M4 were selected as optimal. Interest in them is related to their coverage of three important settings (non-vaccinated, Buck 19-vaccinated and RB51-vaccinated animals). The results of this study indicate a true prevalence of 1.63% in non-vaccinated animals (95% CrI: 0.56–2.54). This estimate is close to a previous estimation of Paucar et al. [36]) that uses the Rose Bengal and SAW-EDTA tests and considers small and medium-scale cattle herds without distinction with regard to the setting and with a true prevalence of 1.6% (95% CrI: 1.0–2.4). The models M3 (S19 vaccinated animals) and M4 (RB51 vaccinated animals) estimate a true prevalence in these settings of 0.97% (95% CrI: 0.005–2.54) and 2.75% (95% CrI: 0.5–5.32), respectively. These true prevalences were significantly different. The results of the present study suggest a decreased true prevalence using the S19 vaccination. Alves et al. [43] have demonstrated that the vaccination with S19 vaccine in 90% of the replacement heifers of 3–8 months of age provides excellent economic returns for the farmers. The results also suggest an increased true prevalence in animals vaccinated with RB51. In contrast, eradication of bovine brucellosis in the Azores (Portugal) based on both test-and-slaughter and mass RB51 vaccine coverage for a sufficiently long period of time was effective to control bovine brucellosis [44]. In Ecuador, vaccination of females (S19 and RB51) is not mandatory, but falls under the responsibility of the farmers [15]. Agrocalidad has standard operating procedures for its use. Commercialized vaccines are registered in Agrocalidad based on the documentation provided by the companies. At this moment, there is no control of quality of the vaccines (e.g., counting of viable cells, determination of the smooth or rough phase of the bacteria as recommended by WOAH) and also no investigation on the level of compliance of vaccination procedures (effectiveness). Therefore, a possible explanation in relation to the observed results in Ecuador is the non-mandatory vaccination (by consequence, the absence of massive vaccination), absence of quality control of commercialized vaccines and the absence of guidelines to estimate the compliance of vaccination procedure. This preliminary finding strongly suggests more investigation on the quality of the vaccination process and a refining of the current national bovine brucellosis control program. To start discussions on improving this control program, we recommend presenting the results of this study to the newly established bovine technical platform in Ecuador, instituted by Agrocalidad and the so called “Mesa Técnica Consultiva Bovina”.

The main limitations of the study were the absence of information about the bacteriological status of each farm investigated and the reliance on specific assay kits.

## 5. Conclusions

In comparison with the cELISA, the results of this study indicate that with similar sensitivity and higher specificity, the iELISA based on innovative synthetic antigen, which is more standardizable should be proposed as a screening test for bovine brucellosis in Ecuador. In addition, the approach proposed gives insight on the quality of the vaccination campaign and claims for refining of the Ecuadorian national brucellosis control program. Follow-up field studies to further investigate vaccination effectiveness, along with a pilot study to implement iELISA in routine screening, are recommended.

## Figures and Tables

**Table 1:** Contingency table showing results of the two diagnostic tests (cELISA and iELISA).

	cELISA		
	Positive	Negative	Total
iELISA			
Positive	a	b	a + b
Negative	c	d	c + d
Total	a + c	b + d	N

Legend: N, total of samples tested by both tests (a + b + c + d).

**Table 2:** Estimates of true prevalence, sensitivity (Se) and specificity (Sp) for competitive (cELISA), and indirect (iELISA) enzyme-linked immunosorbent assays using a Bayesian approach.

Test	Parameter	Prior	Posterior Estimation in Percent (95% CrI)
Model 1 (3299 animals)			
	True prevalence	Uniform [0, 0.1]	1.50 (0.36–2.47)
cELISA	Se	Beta [96, 6]	94.02 (88.56–97.78)
	Sp	Uniform [0.95, 1]	95.85 (95.05–96.76)
iELISA	Se	Beta [96, 6]	94.05 (88.73–97.77)
	Sp	Uniform [0.95, 1]	98.10 (97.03–98.92)
Sensitivity analysis regarding model 1			
	True prevalence	Uniform [0, 0.2]	3.45 (0.24–7.01)
cELISA	Se	Beta [51, 51]	50.27 (40.67–59.85)
	Sp	Uniform [0, 1]	96.08 (94.23–98.05)
iELISA	Se	Beta [51, 51]	49.78 (40.20–59.40)
	Sp	Uniform [0, 1]	98.36 (96.70–99.99)
Model 2 (2506 unvaccinated animals)			
	True prevalence	Uniform [0, 0.1]	1.63 (0.56–2.54)
cELISA	Se	Beta [96, 6]	94.02 (88.60–97.73)
	Sp	Uniform [0.95, 1]	95.64 (95.03–96.49)
iELISA	Se	Beta [96, 6]	94.04 (88.61–97.78)
	Sp	Uniform [0.95, 1]	98.26 (97.29–98.99)
Sensitivity analysis regarding model 2			
	True prevalence	Uniform [0, 0.2]	2.54 (0.17–5.34)
cELISA	Se	Beta [51, 51]	71.18 (51.14–97.92)
	Sp	Uniform [0, 1]	95.66 (93.89–97.69)
iELISA	Se	Beta [51, 51]	73.03 (50.91–98.54)
	Sp	Uniform [0, 1]	96.44 (96.73–99.90)
Model 3 (383 vaccinated animals with S19)			
	True prevalence	Uniform [0, 0.1]	0.97 (0.005–2.54)
cELISA	Se	Beta [96, 6]	94.04 (88.7–97.76)
	Sp	Uniform [0.95, 1]	97.4 (95.57–98.88)
iELISA	Se	Beta [96, 6]3-	94.08 (88.7–97.79)
	Sp	Uniform [0.95, 1]	98.28 (96.56–99.5)
Sensitivity analysis regarding model 3			
	True prevalence	Uniform [0, 0.2]	2.55 (0.16–6.16)
cELISA	Se	Beta [51, 51]	50.06 (40.41–59.73)
	Sp	Uniform [0, 1]	97.87 (95.58–99.7)
iELISA	Se	Beta [51, 51]	49.89 (40.39–59.57)
	Sp	Uniform [0, 1]	98.64 (96.68–99.91)
Model 4 (392 vaccinated animals with RB51)			
	True prevalence	Uniform [0, 0.1]	2.75 (0.50–5.32)
cELISA	Se	Beta [96, 6]	94.01 (88.5–97.77)
	Sp	Uniform [0.95, 1]	96.38 (95.08–98.09)
iELISA	Se	Beta [96, 6]	94.06 (88.69–94.34)
	Sp	Uniform [0.95, 1]	97.33 (95.39–99.02)
Sensitivity analysis regarding model 4			
	True prevalence	Uniform [0, 0.2]	4.21 (0.20–13.02)
cELISA	Se	Beta [51, 51]	54.89 (5.38–97.16)
	Sp	Uniform [0, 1]	95.9 (92.38–99.48)
iELISA	Se	Beta [51, 51]	50.69 (4.03–96.63)
	Sp	Uniform [0, 1]	96.68 (93.47–99.7)

Legend: CrI, credibility interval; Se, sensitivity; Sp, specificity.

**Table 3:** Validity criteria for the four models (M1 to M4) with informative priors and four additional models with non-informative priors used for sensitivity analysis.

Model	Prior	BGR (Mixing Chains)	Bayes p	DIC	pD	Density Distribution Identifiable
M1	Informative	Yes	0.4937	23.923	2.884	More
M1-SA	Non-informative	Yes	0.4926	23.986	2.907	Less
M2	Informative	Yes	0.4918	23.097	2.821	More
M2-SA	Non-informative	Yes	0.5047	23.387	2.965	Less
M3	Informative	Yes	0.5182	16.111	2.583	More
M3-SA	Non-informative	Yes	0.5241	16.282	2.642	Less
M4	Informative	Yes	0.4681	17.675	2.620	More
M4-SA	Non-informative	Yes	0.4909	18.048	2.770	Less

Legend: See Table 2 for details regarding the priors; M1 to M4, models 1 to 4; M1-SA to M4-SA, same models as M1 to M4 but with non-informative priors for prevalence, sensitivity and specificity of both tests; BGR, Brooks–Gelman–Rubin statistic (used to analyze convergence and the degree of mixing of Markov chains); bayes p, Bayesian *p* value; DIC, Deviance Information Criterion; pD, the effective number of estimated parameters.

## Data Availability

The data that support the findings of this study are available from the corresponding author upon request.

## Appendix B

**Table A1:** Conditional probabilities.

Prevalence	*p*(*D*+)	*θ*_1_
Se_1_	*p*(*T*_1_^+^ | *D*^+^)	*θ*_2_
Sp_1_	*p*(*T*_1_^−^ | *D*^−^)	*θ*_3_
	*p*(*T*_2_^+^ | *D*^+^ ∩ *T*_1_^+^)	*θ*_4_
	*p(T*_2_^+^ | *D*^+^ ∩ *T*_1_^−^)	*θ*_5_
	*p(T*_2_^−^ | *D*^−^ ∩ *T*_1_^−^)	*θ*_6_
	*p(T*_2_^−^ | *D*^−^ ∩ *T*_1_^+^)	*θ*_7_

## Appendix C

**Table A2:** Parameters.

*p = θ*_1_
*Se*_1_ *= θ*_2_
*Sp*_1_ *= θ*_3_
*Se*_2_ *= θ*_2_ *θ*_4_ *+ (1 − θ*_2_ *) θ*_5_
*Sp*_2_ *= θ*_3_ *θ*_6_ *+ (1 − θ*_3_ *) θ*_7_

## Appendix D

### Appendix D.1. M1—Code of the Model 1 for All Animals

{

r[1:4] ~ dmulti(p[1:4], n)

p[1] <- pi*(SeElisa*SeSYN+covDp) + (1-pi)*((1-SpElisa)*(1-SpSYN)+covDn)

p[2] <- pi*(SeElisa*(1-SeSYN)-covDp) + (1-pi)*((1-SpElisa)*SpSYN-covDn)

p[3] <- pi*((1-SeElisa)*SeSYN-covDp) + (1-pi)*(SpElisa*(1-SpSYN)-covDn)

p[4] <- pi*((1-SeElisa)*(1-SeSYN)+covDp) + (1-pi)*(SpElisa*SpSYN+covDn)

ls <- (SeElisa-1)*(1-SeSYN)

us <- min(SeElisa,SeSYN) - SeElisa*SeSYN

lc <- (SpElisa-1)*(1-SpSYN)

uc <- min(SpElisa,SpSYN) - SpElisa*SpSYN

pi ~ dunif(0, 0.1)

SeElisa ~ dbeta(96,6)

SpElisa ~ dunif(0.95,1)

SeSYN ~ dbeta(96,6)

SpSYN ~dunif(0.95,1)

covDn ~ dunif(lc, uc)

covDp ~ dunif(ls, us)

rhoD <- covDp / sqrt(SeElisa*(1-SeElisa)*SeSYN*(1-SeSYN))

rhoDc <- covDn / sqrt(SpElisa*(1-SpElisa)*SpSYN*(1-SpSYN))

r2[1:4] ~ dmulti(p[1:4],n)

for ( i in 1:4)

{

d[i] <- r[i]*log(max(r[i],1)/(p[i]*n))

d2[i] <- r2[i]*log(max(r2[i],1)/(p[i]*n))

}

bayesp <- step(sum(d[]) - sum(d2[]))

}

list(r=c(64,117,43,3075), n=3299)

### Appendix D.2. M1-SA—Code of the Model 1 for the Sensitivity Analysis (Non-Informative Priors)

{

r[1:4] ~ dmulti(p[1:4], n)

p[1] <- pi*(SeElisa*SeSYN+covDp) + (1-pi)*((1-SpElisa)*(1-SpSYN)+covDn)

p[2] <- pi*(SeElisa*(1-SeSYN)-covDp) + (1-pi)*((1-SpElisa)*SpSYN-covDn)

p[3] <- pi*((1-SeElisa)*SeSYN-covDp) + (1-pi)*(SpElisa*(1-SpSYN)-covDn)

p[4] <- pi*((1-SeElisa)*(1-SeSYN)+covDp) + (1-pi)*(SpElisa*SpSYN+covDn)

ls <- (SeElisa-1)*(1-SeSYN)

us <- min(SeElisa,SeSYN) - SeElisa*SeSYN

lc <- (SpElisa-1)*(1-SpSYN)

uc <- min(SpElisa,SpSYN) - SpElisa*SpSYN

pi ~ dunif(0,0.2)

SeElisa ~ dbeta(51,51)

SpElisa ~ dunif(0.5,1)

SeSYN ~ dbeta(51,51)

SpSYN ~ dunif(0.5,1)

covDn ~ dunif(lc, uc)

covDp ~ dunif(ls, us)

rhoD <- covDp / sqrt(SeElisa*(1-SeElisa)*SeSYN*(1-SeSYN))

rhoDc <- covDn / sqrt(SpElisa*(1-SpElisa)*SpSYN*(1-SpSYN))

r2[1:4] ~ dmulti(p[1:4],n)

for ( i in 1:4)

{

d[i] <- r[i]*log(max(r[i],1)/(p[i]*n))

d2[i] <- r2[i]*log(max(r2[i],1)/(p[i]*n))

}

bayesp <- step(sum(d[]) - sum(d2[]))

}

list(r=c(64,117,43,3075), n=3299)

### Appendix D.3. M2—Code of the Model 2 for Non-Vaccinated Animals

{

r[1:4] ~ dmulti(p[1:4], n)

p[1] <- pi*(SeElisa*SeSYN+covDp) + (1-pi)*((1-SpElisa)*(1-SpSYN)+covDn)

p[2] <- pi*(SeElisa*(1-SeSYN)-covDp) + (1-pi)*((1-SpElisa)*SpSYN-covDn)

p[3] <- pi*((1-SeElisa)*SeSYN-covDp) + (1-pi)*(SpElisa*(1-SpSYN)-covDn)

p[4] <- pi*((1-SeElisa)*(1-SeSYN)+covDp) + (1-pi)*(SpElisa*SpSYN+covDn)

ls <- (SeElisa-1)*(1-SeSYN)

us <- min(SeElisa,SeSYN) - SeElisa*SeSYN

lc <- (SpElisa-1)*(1-SpSYN)

uc <- min(SpElisa,SpSYN) - SpElisa*SpSYN

pi ~ dunif(0, 0.1)

SeElisa ~ dbeta(96,6)

SpElisa ~ dunif(0.95,1)

SeSYN ~ dbeta(96,6)

SpSYN ~dunif(0.95,1)

covDn ~ dunif(lc, uc)

covDp ~ dunif(ls, us)

rhoD <- covDp / sqrt(SeElisa*(1-SeElisa)*SeSYN*(1-SeSYN))

rhoDc <- covDn / sqrt(SpElisa*(1-SpElisa)*SpSYN*(1-SpSYN))

r2[1:4] ~ dmulti(p[1:4],n)

for ( i in 1:4)

{

d[i] <- r[i]*log(max(r[i],1)/(p[i]*n))

d2[i] <- r2[i]*log(max(r2[i],1)/(p[i]*n))

}

bayesp <- step(sum(d[]) - sum(d2[]))

}

list(r=c(48,99,32,2327), n=2506)

### Appendix D.4. M2-SA—Code of the Model 2 for the Sensitivity Analysis (Non-Informative Priors)

r[1:4] ~ dmulti(p[1:4], n)

p[1] <- pi*(SeElisa*SeSYN+covDp) + (1-pi)*((1-SpElisa)*(1-SpSYN)+covDn)

p[2] <- pi*(SeElisa*(1-SeSYN)-covDp) + (1-pi)*((1-SpElisa)*SpSYN-covDn)

p[3] <- pi*((1-SeElisa)*SeSYN-covDp) + (1-pi)*(SpElisa*(1-SpSYN)-covDn)

p[4] <- pi*((1-SeElisa)*(1-SeSYN)+covDp) + (1-pi)*(SpElisa*SpSYN+covDn)

ls <- (SeElisa-1)*(1-SeSYN)

us <- min(SeElisa,SeSYN) - SeElisa*SeSYN

lc <- (SpElisa-1)*(1-SpSYN)

uc <- min(SpElisa,SpSYN) - SpElisa*SpSYN

pi ~ dunif(0, 0.2)

SeElisa ~ dbeta(51,51)

SpElisa ~ dunif(0.5,1)

SeSYN ~ dbeta(51,51)

SpSYN ~dunif(0.5,1)

covDn ~ dunif(lc, uc)

covDp ~ dunif(ls, us)

rhoD <- covDp / sqrt(SeElisa*(1-SeElisa)*SeSYN*(1-SeSYN))

rhoDc <- covDn / sqrt(SpElisa*(1-SpElisa)*SpSYN*(1-SpSYN))

r2[1:4] ~ dmulti(p[1:4],n)

for ( i in 1:4)

{

d[i] <- r[i]*log(max(r[i],1)/(p[i]*n))

d2[i] <- r2[i]*log(max(r2[i],1)/(p[i]*n))

}

bayesp <- step(sum(d[]) - sum(d2[]))

}

list(r=c(48,99,32,2327), n=2506)

### Appendix D.5. M3—Code of the Model 3 for S19-Vaccinated Animals

{

r[1:4] ~ dmulti(p[1:4], n)

p[1] <- pi*(SeElisa*SeSYN+covDp) + (1-pi)*((1-SpElisa)*(1-SpSYN)+covDn)

p[2] <- pi*(SeElisa*(1-SeSYN)-covDp) + (1-pi)*((1-SpElisa)*SpSYN-covDn)

p[3] <- pi*((1-SeElisa)*SeSYN-covDp) + (1-pi)*(SpElisa*(1-SpSYN)-covDn)

p[4] <- pi*((1-SeElisa)*(1-SeSYN)+covDp) + (1-pi)*(SpElisa*SpSYN+covDn)

ls <- (SeElisa-1)*(1-SeSYN)

us <- min(SeElisa,SeSYN) - SeElisa*SeSYN

lc <- (SpElisa-1)*(1-SpSYN)

uc <- min(SpElisa,SpSYN) - SpElisa*SpSYN

pi ~ dunif(0,0.1)

SeElisa ~ dbeta(96,6)

SpElisa ~ dunif(0.95,1)

SeSYN ~ dbeta(96,6)

SpSYN ~dunif(0.95,1)

covDn ~ dunif(lc, uc)

covDp ~ dunif(ls, us)

rhoD <- covDp / sqrt(SeElisa*(1-SeElisa)*SeSYN*(1-SeSYN))

rhoDc <- covDn / sqrt(SpElisa*(1-SpElisa)*SpSYN*(1-SpSYN))

r2[1:4] ~ dmulti(p[1:4],n)

for ( i in 1:4)

{

d[i] <- r[i]*log(max(r[i],1)/(p[i]*n))

d2[i] <- r2[i]*log(max(r2[i],1)/(p[i]*n))

}

bayesp <- step(sum(d[]) - sum(d2[]))

}

list(r=c(4,7,4,368), n=383)

### Appendix D.6. M3-SA—Code of the Model 3 for the Sensitivity Analysis (Non-Informative Priors)

{

r[1:4] ~ dmulti(p[1:4], n)

p[1] <- pi*(SeElisa*SeSYN+covDp) + (1-pi)*((1-SpElisa)*(1-SpSYN)+covDn)

p[2] <- pi*(SeElisa*(1-SeSYN)-covDp) + (1-pi)*((1-SpElisa)*SpSYN-covDn)

p[3] <- pi*((1-SeElisa)*SeSYN-covDp) + (1-pi)*(SpElisa*(1-SpSYN)-covDn)

p[4] <- pi*((1-SeElisa)*(1-SeSYN)+covDp) + (1-pi)*(SpElisa*SpSYN+covDn)

ls <- (SeElisa-1)*(1-SeSYN)

us <- min(SeElisa,SeSYN) - SeElisa*SeSYN

lc <- (SpElisa-1)*(1-SpSYN)

uc <- min(SpElisa,SpSYN) - SpElisa*SpSYN

pi ~ dunif(0,0.2)

SeElisa ~ dbeta(51,51)

SpElisa ~ dunif(0.5,1)

SeSYN ~ dbeta(51,51)

SpSYN ~dunif(0.5,1)

covDn ~ dunif(lc, uc)

covDp ~ dunif(ls, us)

rhoD <- covDp / sqrt(SeElisa*(1-SeElisa)*SeSYN*(1-SeSYN))

rhoDc <- covDn / sqrt(SpElisa*(1-SpElisa)*SpSYN*(1-SpSYN))

r2[1:4] ~ dmulti(p[1:4],n)

for ( i in 1:4)

{

d[i] <- r[i]*log(max(r[i],1)/(p[i]*n))

d2[i] <- r2[i]*log(max(r2[i],1)/(p[i]*n))

}

bayesp <- step(sum(d[]) - sum(d2[]))

}

list(r=c(4,7,4,368), n=383)

### Appendix D.7. M4—Code of the Model 4 for RB51-Vaccinated Animals

{

r[1:4] ~ dmulti(p[1:4], n)

p[1] <- pi*(SeElisa*SeSYN+covDp) + (1-pi)*((1-SpElisa)*(1-SpSYN)+covDn)

p[2] <- pi*(SeElisa*(1-SeSYN)-covDp) + (1-pi)*((1-SpElisa)*SpSYN-covDn)

p[3] <- pi*((1-SeElisa)*SeSYN-covDp) + (1-pi)*(SpElisa*(1-SpSYN)-covDn)

p[4] <- pi*((1-SeElisa)*(1-SeSYN)+covDp) + (1-pi)*(SpElisa*SpSYN+covDn)

ls <- (SeElisa-1)*(1-SeSYN)

us <- min(SeElisa,SeSYN) - SeElisa*SeSYN

lc <- (SpElisa-1)*(1-SpSYN)

uc <- min(SpElisa,SpSYN) - SpElisa*SpSYN

pi ~ dunif(0,0.1)

SeElisa ~ dbeta(96,6)

SpElisa ~ dunif(0.95,1)

SeSYN ~ dbeta(96,6)

SpSYN ~dunif(0.95,1)

covDn ~ dunif(lc, uc)

covDp ~ dunif(ls, us)

rhoD <- covDp / sqrt(SeElisa*(1-SeElisa)*SeSYN*(1-SeSYN))

rhoDc <- covDn / sqrt(SpElisa*(1-SpElisa)*SpSYN*(1-SpSYN))

r2[1:4] ~ dmulti(p[1:4],n)

for ( i in 1:4)

{

d[i] <- r[i]*log(max(r[i],1)/(p[i]*n))

d2[i] <- r2[i]*log(max(r2[i],1)/(p[i]*n))

}

bayesp <- step(sum(d[]) - sum(d2[]))

}

list(r=c(12,11,7,368), n=392)

### Appendix D.8. M4-SA—Code of the Model 4 for the Sensitivity Analysis (Non-Informative Priors)

{

r[1:4] ~ dmulti(p[1:4], n)

p[1] <- pi*(SeElisa*SeSYN+covDp) + (1-pi)*((1-SpElisa)*(1-SpSYN)+covDn)

p[2] <- pi*(SeElisa*(1-SeSYN)-covDp) + (1-pi)*((1-SpElisa)*SpSYN-covDn)

p[3] <- pi*((1-SeElisa)*SeSYN-covDp) + (1-pi)*(SpElisa*(1-SpSYN)-covDn)

p[4] <- pi*((1-SeElisa)*(1-SeSYN)+covDp) + (1-pi)*(SpElisa*SpSYN+covDn)

ls <- (SeElisa-1)*(1-SeSYN)

us <- min(SeElisa,SeSYN) - SeElisa*SeSYN

lc <- (SpElisa-1)*(1-SpSYN)

uc <- min(SpElisa,SpSYN) - SpElisa*SpSYN

pi ~ dunif(0,0.2)

SeElisa ~ dbeta(51,51)

SpElisa ~ dunif(0.5,1)

SeSYN ~ dbeta(51,51)

SpSYN ~dunif(0.5,1)

covDn ~ dunif(lc, uc)

covDp ~ dunif(ls, us)

rhoD <- covDp / sqrt(SeElisa*(1-SeElisa)*SeSYN*(1-SeSYN))

rhoDc <- covDn / sqrt(SpElisa*(1-SpElisa)*SpSYN*(1-SpSYN))

r2[1:4] ~ dmulti(p[1:4],n)

for ( i in 1:4)

{

d[i] <- r[i]*log(max(r[i],1)/(p[i]*n))

d2[i] <- r2[i]*log(max(r2[i],1)/(p[i]*n))

}

bayesp <- step(sum(d[]) - sum(d2[]))

}

list(r=c(12,11,7,368), n=392)

## Appendix E

**Table A3:** Distribution of parameters for models M1 to M4.

Parameter	M1 (All Animals)	M2 (Non-Vaccinated Animals)	M3 (S19-Vaccinated Animals)	M4 (RB51-Vaccinated Animals)
Sensitivity of the cELISA				
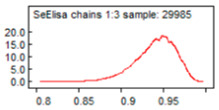
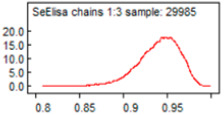
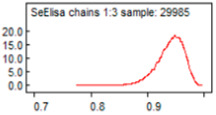
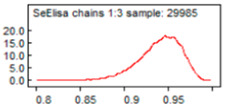
Sensitivity of the iELISA				
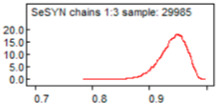
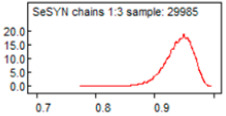
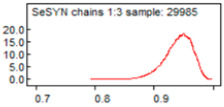
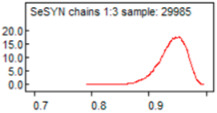
Specificity of the cELISA				
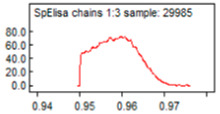
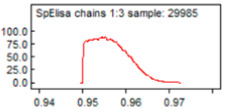
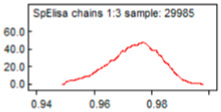
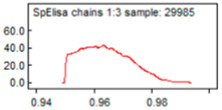
Specificity of the iELISA				
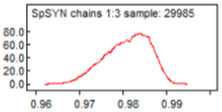
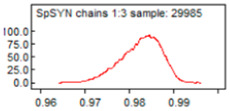
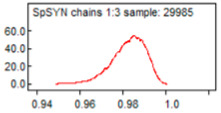
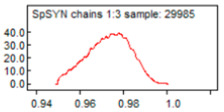
True prevalence				
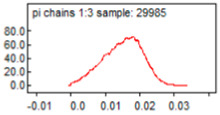
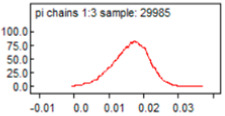
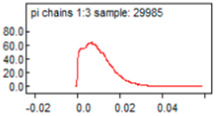
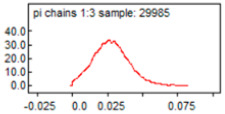

Legend: Elisa as cELISA; SYN as iELISA; Se as sensitivity; Sp as specificity; pi as true prevalence.
